# The Medication Experience: A Concept Analysis

**DOI:** 10.3390/pharmacy9010007

**Published:** 2020-12-31

**Authors:** Lisa A. Hillman, Cynthia Peden-McAlpine, Djenane Ramalho-de-Oliveira, Jon C. Schommer

**Affiliations:** 1 College of Pharmacy, University of Minnesota, Minneapolis, MN 55455, USA; schom010@umn.edu; 2 School of Nursing, University of Minnesota, Minneapolis, MN 55455, USA; peden001@umn.edu; 3 College of Pharmacy, Universidade Federal de Minas Gerais (UFMG), Belo Horizonte, MG 31270-901, Brazil; droliveira@ufmg.br

**Keywords:** medication experience, patient-centered care, pharmacist, concept analysis

## Abstract

This is a concept analysis of the medication experience with a focus on how it applies to the pharmaceutical care practice framework used by pharmacist practitioners. The medication experience is a vital component of pharmaceutical care practice and of patient-centered care. Although the experience of taking medication has been studied across disciplines for decades, a concept analysis of the medication experience is lacking. Rodgers’ evolutionary concept analysis method was utilized. Ovid Medline, CINAHL, PsycINFO, Sociological Abstracts and Google Scholar databases, references and hand searches were used to compile an international dataset of 66 papers published from 1982 to 2020. As a result of the available literature, the medication experience is defined as one of ambivalence and vulnerability in which the patient is actively engaged in an ongoing process or negotiation, which is pragmatic to the ways in which they live and experience life, contextualized and nuanced within the social construction of their individual realities. The concept of medication experience is an important addition to the scientific literature. The definition of medication experience from the perspective of the patient will help to better explain the concept for future research and theory development to move the discipline of pharmaceutical care practice forward.

## 1. Introduction

The medication experience, or the subjective experience of taking medications from the perspective of the individual user of the medication, has been explored for decades in pharmacy and across multiple disciplines. While facets of the lived experience of medication-taking were being explored in the 1980s and 1990s, to our knowledge, no concept analysis has been done to clarify its various dimensions and develop an interdisciplinary definition of the concept of the medication experience to be utilized in the context of pharmaceutical care practice. Previous work in pharmacy has established the medication experience as an important practice concept for pharmaceutical care and provided a definition applicable to chronic conditions [1,2,3]. For this concept analysis, we looked across disciplines to generate a broader definition and characteristics of the medication experience.

Pharmaceutical care is a patient-centered practice defined in the 1990s and has been instrumental to the shift in the profession of pharmacy from product-focused to patient-centered [4,5,6]. Optimizing medication therapy for patients so that it is both safe and effective while also acceptable to the patient is an urgent healthcare priority [4,5,6]. The pharmaceutical care practitioner is the professional in society best suited to fill this societal need. This practitioner accompanies the patient in his/her process of taking medications in daily life in a manner that acknowledges the patient’s wishes, expectations, concerns, and life experiences, besides the use of all technical evidence. The medication experience is at the heart of pharmaceutical care and is key to what makes it patient-centered [7]. 

To be patient-centered is to have the patient’s interest taken into account during all aspects of the patient care process. The medication experience forms the context for the activities performed by the pharmacist in identifying and resolving drug therapy problems and patient counseling [2,3]. In contrast to the biomedical perspective on medication adherence, which expects the patient to follow the directions and recommendations of healthcare professionals, the lived experience perspective provides an alternative paradigm to viewing patients’ attitudes and behaviors regarding medication use. From this perspective, the patient’s behaviors happen in the context of their lived experience with their health conditions and all the meanings they ascribe to taking medications in daily life. Patients’ life circumstances, feelings, previous experiences, and bodily experiences can affect their perspectives and behaviors around medications. Thus, understanding how the individual patient relates to her/his conditions and medications is crucial to a patient-centered practice [4,5,6].

The purpose of a concept analysis is to add to the development of knowledge. Concepts are discussed as the building blocks necessary to construct a theory or a theoretical framework. In the literature on research methods, the conceptual basis of a research study provides strength to any investigation. The philosophical literature discusses what constitutes knowledge, and concepts are a major component of these discussions. Concepts are disciplinary in nature and are a reflection of the ways in which we understand and express ideas within disciplines. Concept analyses provide a conceptual definition synthesized from the literature with its defining attributes, antecedents, and consequences [8].

A well-developed concept can help to build knowledge that is useful and practical when applied to the provision of care, theory development, and research. The purpose of this analytic review of the extant literature is to provide the language and a theoretical foundation to the important concept of the medication experience. The authors acknowledge that several disciplines have a stake and interest in medication-taking from the perspective of the patient. Some have done considerable research in this domain, ahead of the practice of pharmacy’s movement into the patient care arena in the 1990s. Given these contributions, the disciplines of medicine, nursing, psychology, and sociology were included in this review of the literature for analysis and contribution to this important knowledge. 

## 2. Materials and Methods 

### 2.1. Description of Evolutionary Perspective of Concept Analysis

While several approaches exist in the analysis of a concept of interest, the evolutionary approach as described by Rodgers was particularly well suited for the stated purpose. As the name implies, the evolutionary approach accounts for the temporal perspective of an evolving concept as more research is completed and shifts occur that contribute to its usefulness in practice and research. It is an inductive technique that is consistent with the way in which knowledge is produced and theory developed [8]. This is relevant for the medication experience as both the concept itself and the profession of pharmacy are in the midst of rapid change in our society since the use of medicines as the mainstay of treatment has grown dramatically [9]. 

A concept by definition is a cluster of attributes. A concept analysis consists of identification of a concept of interest including surrogate terms, a setting for sample selection, collection of data relevant to attributes of the concept, contextual basis of the concept that is inclusive of antecedents and consequences as well as interdisciplinary and sociocultural variations, analysis of data according to these characteristics, identification of an exemplar, and implications for further development of the concept. The attributes are the characteristics that describe the common aspects or characteristics of the phenomenon. Antecedents are the conditions that lead up to the concept of interest while the consequences define what happens as a result of the concept of interest. Surrogate terms are those terms in the literature that have similar meaning to the concept of interest and are often used interchangeably but have been named differently. Related concepts have overlying similarities and share a relationship with the concept but do not share the same set of attributes [8].

### 2.2. Data Sources and Sample Selection

The databases of Ovid Medline, PsycINFO, CINAHL, Sociological Abstracts, and Google Scholar were systematically searched with the experienced assistance of the University of Minnesota librarians from Nursing and Pharmacy to allow for the broadest inclusion of articles. No time limits were applied to leave open the possibility for earliest use of the term and concept. Articles were limited to English. From an epistemological standpoint, the knowledge of interest here is not the clinician/expert bias that is steeped in the biomedical model, but from the patient themselves. Therefore, the inclusion criteria for this concept analysis were limited to the perspective of the patient taking medications. Exclusion criteria included the healthcare provider point of view on medication experience, experiences of patients who were hospitalized, in palliative and end of life care, and children and adolescents. While the medication experience is relevant in any setting in which a person takes medications, including institutions such as hospitals and long-term care facilities, for this analysis, articles were limited to the ambulatory setting to obtain the patient perspective as close as possible to the natural setting and to keep the study manageable and focused.

Due to the inexactness in terminology used to refer to the medication experience in the literature, three searches were undertaken. These were: (1) medication and medicines experience, (2) qualitative + medication + patient experience, and (3) meaning of medications. The “*” notation allowed for the inclusion of all variants of the indicated search term. Please refer to Table 1 for details on search terms within each search. 

**Figure 1:**
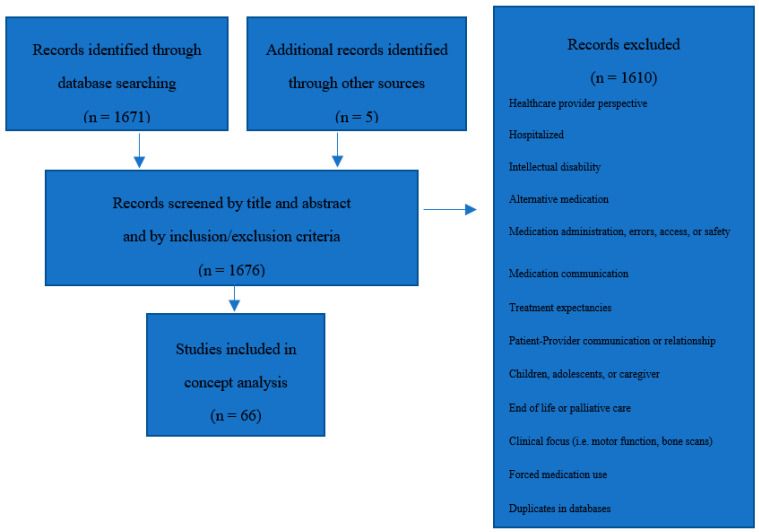
Sample selection process.

The total search results were 1671 articles. To arrive at the most suitable articles to include, two authors (LAH and DRO) reviewed the first 84 titles and abstracts together to come to agreement on inclusion/exclusion criteria and identify additional search strategies if needed. For example, if the main focus of the study was not about the medication experience but about a related topic such as coping, medication errors, or medication safety, these were excluded easily by title and abstract. In addition, the searches of medicines experience and meaning of medication were included as search strategies. A total of 61 articles were identified after the review of titles, abstracts, and texts. An additional five articles on the topic were included from references of other articles and deemed by the authors to be relevant to the search (see Figure 1). 

Articles included 49 qualitative research studies [10,11,12,13,14,15,16,17,18,19,20,21,22,23,24,25,26,27,28,29,30,31,32,33,34,35,36,37,38,39,40,41,42,43,44,45,46,47,48,49,50,51,52,53,54,55,56,57,58], six articles of instrument development and psychometrics [59,60,61,62,63,64], four meta-ethnographies [1,65,66,67], five qualitative systematic literature reviews [68,69,70,71,72], one research note of interim data [73], and one personal case study [74]. Countries were represented from Australia, Brazil, Cambodia, Canada, Denmark, Germany, Iran, Japan, the Netherlands, New Zealand, Spain, Sweden, Switzerland, Thailand, the United Kingdom, the United States of America, and Wales. Chronic disease context in studies is broadly categorized as follows: (1) specific conditions that include AIDS/HIV, schizophrenia or schizoaffective disorder, anxiety and/or depression, serious mental illness, osteoporosis, type 2 diabetes mellitus, multiple sclerosis, rheumatologic conditions, epilepsy, asthma, Hepatitis C, coronary heart disease, stroke, hypertension, post-MI secondary prevention, idiopathic pulmonary fibrosis/kidney transplant/hemolytic uremic syndrome; (2) specific medications that include oral chemotherapy, opioids, benzodiazepines, antipsychotics, anti-depressants, statins for primary CVD prevention, insulin for gestational diabetes mellitus; (3) population types that include older adults with chronic conditions, African Americans, Puerto Ricans, community mental health users, and users of multiple medications for chronic conditions. Research articles that were included were assessed for quality based on the Lincoln and Guba criteria of credibility, confirmability, dependability, authenticity, and transferability [75]. Three authors (LAH, DRO, JCS) assessed each criterion and how it was met in each study. All authors were in agreement on final assessment of quality of articles included in the concept analysis. 

### 2.3. Data Analysis

A Microsoft^®^ Excel table was constructed to identify references, attributes, antecedents, consequences, related concepts, surrogate terms, and specific contexts using Rodgers evolutionary analysis procedures. Analysis followed an inductive approach to identify these elements that were then synthesized into a comprehensive definition. The analysis focused on what is common in the use of the concept. Statements that provided insight to answer the question “what are the characteristics of the medication experience?” were used to determine elements [8]. Initially, one author (LAH) was directly involved in data analysis. Word labels were selected that provide clear descriptive categories of the elements of the concept analysis using actual (in vivo) words from the text. A small sample represented by the pharmacy discipline was initially reviewed and analyzed by LAH. This initial coding scheme was shared with CPM and DRO to determine validity of codes. Authors met to discuss and resolve any discrepancies and arrive at consensus. This process was repeated with the remainder of the pharmacy articles and then the articles represented by the other disciplines. All elements were reviewed for content, coherence, and agreement among authors, and agreement was reached. Throughout this process of analysis and consensus discussions, the 4th author (JCS) was included on all analysis materials and discussion points to provide additional consensus. All analysis materials were accessible to authors via Google Shared Drive^®^. 

## 3. Results

The results will be presented in text summary under the headings of: Surrogate Terms and Related Concepts, Attributes, Antecedents and Consequences, and the Definition of the Medication Experience. Table 2, Table 3 and Table 4 are included throughout this section as a summary of the text and to assist in orienting the reader to the specific ideas presented. 

### 3.1. Surrogate Terms and Related Concepts

Surrogate terms refer to concepts that are synonymous and interchangeable. Surrogate terms for medication experience include “Medication-taking experiences” [12], *“*Medicines use” [71], “Medication taking practices” [12], “Medication practice” [23], “Meaning of medications” [11], and “Medication-related experiences” [59].

Related concepts are concepts that share relationships but not the same attributes as the medication experience. These include “Medication-related needs” [66], “Medication taking behavior” [69], and “Medication adherence” [55].

### 3.2. Attributes

**Ambivalence.** The medication experience puts an individual in a state of ambivalence. This state implies that a person has simultaneous and contradictory attitudes or feelings toward something. For instance, some patients perceive medications as a necessary evil [32,44,69] while others want to avoid medications but recognize the need to take medications [40,42,69,73]. There may be both gratitude for the potential benefits along with anxiety about side effects and general skepticism about net gain [11,25,27,49,59], leaving patients to weigh the risks and benefits of taking medications [18,29,48]. It is a potential conflict that can present in different forms at different times, depending on the context of the situation and on the risks and benefits involved from the perspective of the person taking the medication. This resistance may be due to the medications themselves or their preferences to use medications [14,17,70]. Given patients’ ambivalence to take medications, they may not take important medications that can support their health.

**Vulnerability.** Descriptions from patients that elicit feelings or concerns demonstrate the vulnerability that they perceive about taking medications. Taking medications puts individuals at risk for the actual and perceived effect of drugs on the body [51,58]. This can be an actual physical harm from side effects of medications [22,46] or from their potential to cause harm [49,54,59], either historical or present [63], leaving patients in a state of “disquiet” [58] or emotional distress [66,68] about medication use. Moreover, they describe vulnerability as their perceived risk of taking a drug whose effectiveness is uncertain for them while living with the potential risk of significant side effects [15,47,51]. For patients who may need to use medications chronically, this long-term timeframe can elicit fear and a mix of emotions related to long-term use and consequences [17,21,62,72] as well as the fears related to the industrialized or synthetic nature of medications, as compared to natural products [17,42]. Additionally, patients are susceptible to the possibility of drug shortages and their high cost [25,42].

Furthermore, the medication experience involves the patient’s reliance on and trust in the healthcare system and its providers. Not only do providers allow access to medications, but patients also need more information to feel comfortable about medication use [12,15,26,30,31,42,67]. Patients need a trusting relationship [27,36,39,40,66] with providers to engender a sense of security about their medications [11,44,52,72]. They depend on pharmacists and providers to give them accurate and understandable information about the effects and side effects of the drug and provide continuity and follow-up care to ensure safety and confidence [49,50,54,66]. This dependence leads to a sense of vulnerability while needing to take medications for one’s health.

**Socially constructed**. The medication experience does not represent a biological or a natural meaning, but rather it is shaped by shared and accepted social and cultural ideas that define individual realities. How one sees reality is subject to a variety of historical, social, and cultural influences that can change over time. This allows for the existence of perspectives outside of the biomedical model, for example, when it comes to the experience of taking medications. What is understood to be “normal” by one individual may vary greatly from that of another. 

One common way that medications can be viewed as socially constructed within the medication experience is their association with symbols or social objects. From the patient’s perspective, medications represent a visible aspect of healthcare or of an underlying illness [42]. They can be symbolized as a reminder of a disease or ill health, of losing control over one’s deteriorating health, or as not normal or healthy [10,23,38,42,51,52]. These notions can result in feelings of shame of personal failing [16,45,67] and feeling bad about taking too many medications [43]. Dependence on medications influences a person’s sense of self [13,56,72] and their independence and functioning. This reinforces the perception that they are not the person they once were [11,32]. These ideas can lead to feelings of social stigmatization [23,33,45,56], such as diabetics being frail and no longer fit [12]. On the other hand, taking medications can be experienced as normalizing life or as commonplace in people’s lives [1,42]. Finally, alternative natural supplements are perceived as safe [34] while medications are not natural but composed of chemicals that may cause side effects that lead to suffering. 

Additionally, what has happened with friends and family while taking medications influences a person’s perspective of what it means to take such medications, and what might happen to them if they take them [16,24,47] can also influence one’s emotional response to illness [34,51]. The media influences the perceived safety of medications, how effective medications actually are, and what should matter as an outcome to an individual taking a medication [22,24,41,46]. Beliefs associated with these social systems all shape an individual’s reality and thus what the medication experience may mean to them. 

Finally, healthcare provider perspectives, rooted in biomedicine, are a powerful social influence when it comes to meaning formation and what is perceived as normal about medication use. Patients hold beliefs that their personal experiences and perspectives are legitimate [24,46,47]. However, in the clinical setting, the authority one has over one’s own lived experience may not be considered as legitimate as the biomedical knowledge possessed by the clinician. The dual nature of the personal perspective and biomedical perspective is a frequent tension within the patient’s medication experience. For example, from the lens of the provider, the term “control” can refer to long-term health outcomes and symptom control, while within a person’s day to day life, “control” may mean the ability to live according to one’s own wishes and their perceived quality of life [21,72]. In Deegan’s (2007) personal account of medication use, she provides both her perspective as a patient as well as that of the biomedical model [74]. Table 2 describes some examples of these tensions that she discusses that arise from the difference in biomedical definition of terms and the patient perspective in applying the use of these terms in their daily life. 

**Pragmatic.** The medication experience involves dealing with medication use sensibly and realistically in a way that is based on practical rather than theoretical considerations. People move through their lives, making decisions and having experiences in a pragmatic and concrete manner. The priority is on wanting to feel well from their perspective, getting on with life and having hope [27,30,40,54]. Patients focus on practical barriers to everyday living such as side effects, disruption to routines, and the practicalities of medication use, such as accessing the medication and service issues, packaging, and administering the medication [11,16,28]. In order to learn about their disease and medications, they seek information that is meaningful to them and is inclusive of many sources, including their own experience [19,24,34,48,69]. Family and friends are often a more pragmatic source of information than healthcare providers that are not as easily accessible physically and whose language may also not be easy to comprehend [13]. Furthermore, their personal evaluation of diagnosis, symptoms, effectiveness, and side effects is often practical and based on tools available to them, such as ability to perform daily activities, not feeling sick, or solving a problem to the physical body [10,21,28,29,42,53]. If they feel the same as before, medications may be perceived as irrelevant or not bad for them in any way [44,68]. 

**Contextual and nuanced.** Contexts are the circumstances that form the setting for an event, statement, or idea and in terms of which it can be fully understood and assessed. The medication experience depends on the specific context of any number of variables such as health and illness experience, specific medications and past use, symptoms, social and daily life circumstances, personal beliefs, attitudes, or one’s desire for involvement in care. Specific medications as well as overall burden of medication use form the context in which medication use is experienced. For example, different medications hold different importance depending on one’s own evaluation and experience [29,54]. There may also be the perception of taking too many medications [44,55], the burden of polypharmacy [62], or one’s past experiences with medication use and side effects [57], all of which influence the medication experience. In addition, how one experiences health and illness influences how the use of medication is situated in the overall health and wellness process [11,14,35,36,54,55]. The context in which one receives health assessment and information may differ when more than one provider is involved [22] while clinic wait times, the type of environment, and attitudes of providers also have an influence on patients’ perspectives [32,36,44], as does one’s relationship with providers [12,59]. 

Social and daily life circumstances as well as personal beliefs and preferences are additional contexts in which medication-taking occurs that impact one’s medication experience. Daily life circumstances that can change regularly or over one’s life are important contexts that account for variation between individuals taking medications [32,55] but can also vary within the same individual over a period of time [20,53,61]. For example, one may have stresses in life [47], such as caring for others, financial constraints [59,68], or of being cared for oneself by a caregiver [50,60]. For an individual, beliefs and general attitudes concerning how and when medications should be effective [65,67] and how easily one may accept the need to take medications [44] are additional contexts to consider. The informational context within which one receives information, such as from family, the media, or the healthcare system, also provide context to consider when understanding one’s medication experience [13,24,34,39,73]. This context will also vary over time as one gains additional information about their particular medications in each specific individual’s circumstance and their readiness for information. 

**Active ongoing process.** The medication experience is a series of actions that occurs over time and requires effort. The medication experience is a process of resistance and acceptance of one’s need to take medications, which may or may not be a struggle [18,43,44,52,55,68]. Patients may have an emotional response to illness or medications that can be strong at first but may lessen over time with acceptance [34,50,67]. Adaptations to daily life and routines, learning to think of medicines and health in a new way, and learning to relate to and cope with side effects and feelings of insecurity and inconvenience [25,28,40,60] are all active processes that the medication experience engenders [44]. Even someone resisting medications, denying the need for medications, or who is a passive consumer of healthcare and medicine is actively evaluating reasons to take the medication, or not, based upon their specific situation [65]. Some people deal with feelings of doubt that they have a disease [16,47], determine on their own when medications are needed [23,42], and self-adjust medications to manage adverse effects or dissatisfaction [19,45]. Multiple forms of patient burden and challenges inherent in the medication experience [49,66] have been reported. They must determine when to seek help and then navigate the healthcare system [22]. Management of daily medications must be accepted with sometimes complex medication regimens that interfere with daily life [62] and require new daily habits [16]. Personal responsibility is required for management of side effects [44] as well as stigma about disease and medications, and often information overload [18,62]. The long-term impact of medications is a reminder that the medication experience is continuous and changes over time [20,34,38,42,47,71].

### 3.3. Antecedents

Antecedents represent the conditions that precede the medication experience, and consequences represent the outcome of having been subject to the medication experience. Both antecedents to and consequences of the medication experience are somewhat implicit in the literature.

One must be a user of medications. In addition, most people have past experiences that influence a belief system about health, illness, and themselves that precedes and shapes an orientation to medications and precedes the medication experience. In other words, a person does not enter into the medication experience with a “blank slate”. Rather, a patient comes to the experience with socially constructed ideas that become pragmatically a part of one’s present life [65,73].

### 3.4. Consequences

The consequences of the medication experience are (1) a reconceptualization of one’s self and (2) a new reality as one learns to live with taking medications, or not, as a result of knowledge steeped in experience. People with illness who take medications have a new understanding of what their health means to them, what they are willing to live with, and what gives them hope. Medication-taking behaviors such as medication adherence are consequences of the medication experience [51].

With all these elements in mind, the following definition for the medication experience was arrived at. 

### 3.5. Definition of Medication Experience

As a result of the available literature, the medication experience is defined as an experience of ambivalence and vulnerability in which the patient is actively engaged in an ongoing process or negotiation, which is pragmatic to the ways in which patients live and experience life, contextualized and nuanced within the social construction of their individual realities.

Table 4 presents an exemplar of the attributes of the medication experience in order to provide a practical representation of the concept in context [8].

## 4. Discussion

This concept analysis, based on Rodgers’ evolutionary approach, contributes a new way to make sense of the medication experience by providing an interdisciplinary definition that can impact theory development, future research, and implications for practice. With use of specific language to define attributes, or characteristics, we are able to formulate an understanding of the medication experience as it is lived by patients. Attention to the interplay of the attributes of ambivalence, vulnerability, the socially constructed and contextual and nuanced nature of a pragmatic, active ongoing process, help to develop a broader and more practical understanding of this phenomenon. First, this discussion will focus on implications of this concept analysis for theory development by discussing the attributes and related literature within the pharmaceutical care model that is foundational to pharmaceutical care practice. The second section of the discussion identifies areas for future research using the definition and attributes from the medication experience concept analysis. The last section of the discussion applies knowledge of the attributes to the practice of pharmacy.

### 4.1. Theory Development

The concept analysis gives form to each of the three core principles of the pharmaceutical care model, particularly as each is grounded in patient-centeredness. According to Cipolle, Strand, and Morley (2012), pharmaceutical care practice, as with other healthcare professions, has three main components: a philosophy of practice, a patient care process and a practice management system [6].

#### 4.1.1. I. Philosophy of practice

The philosophy of pharmaceutical care practice represents the foundation of the practice and includes four key elements that can be influenced by the patient’s medication experience. The four key elements are:

(a) The *description of the social need for the practice* is to “optimize the use of medications and minimize the drug-related morbidity and mortality” related to drug use [6] (p.77). The related attribute is **pragmatic** in nature from the patient’s perspective. What patients want and expect is defined in ways meaningful and visible to them. The optimization of medications is dependent upon how the patient views and feels about taking medications. Hence, pharmacists need to reframe their assessment to focus on the pragmatic and concrete nature of the patient’s medication experience to better understand the meaning of their medication experience that will provide quality pharmaceutical care.

The attribute of **ambivalence** in the process of taking medications presents the context in which the patient makes an initial decision that often includes both caring about one’s health and not wanting to take medications. Pound et al. (2005) describe how the resistance held by patients is toward the medications themselves and their concerns of safety [70]. In addition, the attribute of **vulnerability** has implications for the patient’s perspective of the effectiveness and safety of taking their medications. Vulnerability may be expressed as fear and uncertainty about what taking medications means for one’s health and the physical effects that medications can cause to one’s body [51]. White et al. (2013) discovered that patients hold concerns about medications that are unrelated to whether or not they adhere [58]. The implications for pharmaceutical practice related to the attribute of ambivalence suggest that pharmacists need to assess the meanings of possible vulnerabilities that the patient may be experiencing. Identifying fear and uncertainty about taking a medication may lead to appropriate changes in a patient’s care plan that have implications for adherence.

(b) The *clear statement of individual practitioner responsibilities to meet this social need* is to identify, resolve, and prevent drug therapy problems during the patient care process to ensure that all drugs are indicated, effective, safe, and convenient [6].

To identify, resolve, and prevent drug therapy problems, the patient’s medication experience must be elicited and assessed throughout the patient care process. It is within the medication experience that the clinician must apply the rational decision-making process (the assessment of indication, effectiveness, safety, and convenience for each medication, in this particular order) [6,78,79]. The attributes of **pragmatic** and **contextually nuanced** inform the practitioner about how patients understand the need for medications (Indication), how patients evaluate if and how they are working (Effectiveness), if the risks incurred are acceptable to them (Safety), and if the medications have a fit, in very concrete ways, with their daily routines (Convenience). In his article describing the self-regulating behaviors of patients with epilepsy, Conrad (1985) demonstrates the practical approach that patients take towards their medication [23]. Further, it has been shown that the context of disease [69], social context such as aging [13], or past illicit drug use [55], or of daily life circumstances [17,47] all have implications for a patient’s medication experience. Pharmacists need to consider the patient’s meaning of the medication experience in terms of the pharmacotherapy work-up. It is crucial that these pragmatic and contextual perspectives of the patient be incorporated into the decision-making process.

(c) There is *the expectation to be patient-centered*, which means “the practitioner will start with the patient’s needs and provide care until they are all met” [6] (p.79).

The expectation of patient-centered care must include knowledge of the patient’s medication experience. The attributes of **pragmatic** and **active ongoing process** are critical to assessing the patient’s perspective. Patients must be actively involved in constructing their medication experience and do so pragmatically. Donovan and Blake (1992) showed that patients’ own perceptions and personal and social circumstances were key considerations in understanding medication-taking behavior [24]. How patients interpret and evaluate their health and lived experience is legitimate knowledge that is imperative to the clinician’s patient-centered approach [80]. The patient’s evaluation of their medication experience is an active, ongoing process as they continue to gather new information and gain new knowledge about the role of medications in their daily lives, the impact on their bodies, and what it means to accept taking medications [39,50]. Thus, the pharmacist must consider that a patient’s medication experience is an ongoing process that must be continuously evaluated.

(d) The *requirement to function within the caring paradigm* means that practitioners “take the time and energy to understand each patient’s medication experience so we can optimize future experiences with drug therapy” [6] (p.47).

The caring paradigm recognizes the necessity of a therapeutic relationship that involves respect and trust [6,7]. The attributes of **socially constructed** and **ambivalence** are important to consider in the patient care process and pharmacotherapy work-up. One important point is the consideration of social and cultural ideas that shape individuals’ realities. Because of cultural influences, what one patient thinks is normal or acceptable may vary greatly between patients with different cultural lenses [11,69]. For instance, people who have lived their lives in the US and people who have come to live in the US from other countries may have different beliefs and values about medications that must be considered. In addition, there is a social and relational aspect to the process in which a patient engages in order to become familiar with one’s medications [71]. When providing culturally sensitive care for a patient, pharmacists must recognize social and cultural influences and integrate them into a unique patient’s assessment, care plan, and follow-up.

**Ambivalence** is also a common experience among those who take medications. Ambivalence itself is a socially constructed aspect in that medications may be culturally accepted in one community and not in another. Furthermore, for the individual living within a particular social and cultural system, while medications may be socially accepted, patients may still hold meanings about health, wellness, and one’s sense of self that must be dealt with. For example, there may be a conflict between the potential benefits of taking the medication and the anxiety about its side effects [37] or other conflicting social priorities that impact medication-taking [16]. Alternatively, the medication may remind them of an illness that they are not yet willing to accept [10]. Pharmacists need to consider these values and beliefs in light of any ambivalent feelings when developing goals and interventions that may positively influence current and future experiences with drug therapy.

#### 4.1.2. II. Patient Care Process

The patient care process is “what the patient experiences when he or she receives pharmaceutical care” and reflects the work between patient and practitioner [6] (p.49).

Practitioners must be tuned into the medication experience throughout the patient care process for a patient to feel cared for and that the service received is valuable to them. The attributes of **vulnerability** and **active ongoing process** in the concept of medication experience are important aspects in the development of a trusting relationship. The **vulnerability** of patients’ must be considered in order to form a trusting, empathic, and therapeutic relationship to engender a sense of security. Thus, pharmacists must give them accurate and understandable information about the medication and provide continuity of care to build a trusting relationship so that the patient feels safe and confident. The attribute of **active ongoing process** informs the pharmacist about the burden and the work that taking medications entails [66]. The pharmacist must be attentive to the emotional aspects and the work that the patient engages in as part of the medication experience in order to gain understanding through follow-up over time and incorporate this knowledge into care plan development.

#### 4.1.3. III. Practice Management System

Practice management systems are “all the resources required to provide a service to patients in an effective and efficient manner” that includes a clear mission that defines the environment and culture of the practice and recognition of tangible and intangible resources required [6] (p.57).

Attention to the medication experience and the attribute of **socially constructed** is important to the practice management system. For pharmacists, it is critical to plan, implement, and manage effective comprehensive medication management (CMM) services to serve patients, each with their unique social and cultural needs. Preparedness of the practitioner is thus the most important requirement to establish a successful practice management system based on a culture of care. While the practitioner must stay competent in therapeutics, and remain committed to the mission of identifying, resolving, and preventing drug therapy problems, the imperative and possibly the most challenging work will be staying focused on the patient’s medication experience. Pharmacists need to be well prepared with knowledge of illness and the contextual aspects of the patient’s medication experience to identify the necessary skills to create treatment strategies to implement in practice.

A specific kind of organizational culture, one that is caring in nature, will be crucial for the patient’s medication experience to emerge, be noticed, and be used in caring for the patient. This caring culture must be built among and shared by a diverse team of interdisciplinary professionals. Knowledge of the medication experience can serve as common language not only in the care of patients directly but as part of a team built on common principles of care.

### 4.2. Future Research

Future research can be informed by this concept analysis on the medication experience given the interdisciplinary definition and attributes generated from this work. This definition will help future researchers to clarify the application of the attributes of the medication experience in different contexts with diverse groups of patients. Future qualitative study designs will allow researchers to study the medication experience in different contexts and cultures and understand the unique medication experiences of patients that will inform practice. From these studies, researchers can develop and refine instruments to deepen the understanding of the medication experience. The definition and attributes derived from this concept analysis can be instructive in instrument development that reflects the general experiences of patients across disease states, revealing the complexities associated with patients’ sociocultural realities.

This concept analysis provides an initial direction for the development of a theory of the patient’s medication experience that can further inform patient-centered pharmaceutical care practice. The new definition and the attributes of the medication experience provide a foundation to develop concepts and their interrelationships foundational to theory development. A theory of the patient medication experience can direct hypothesis development for testing and subsequent research to expand the theory.

### 4.3. Pharmaceutical Care Practice

This concept analysis provides a new approach to the definition of the medication experience that expands its utility as a practice concept, thereby improving the possibility of true patient-centered care. When defined by the patients themselves, inclusion of the medication experience by providers can lead to a more powerful shared decision-making process that keeps the care central to the patient, makes it more authentic, and strengthens patient–provider relationships. However, practitioners need to be able to access the medication experience in a systematic way. Language that is specific to the medication experience as defined in this analysis can be used to create space for specific kinds of conversations to happen, empathy to be experienced, and trust to be built. Practically speaking, knowledge of these attributes of the medication experience suggests questions that practitioners can use to gain access to this patient experience. Thus, Table 5 provides some directions on the type of questions that practitioners might pose to patients to gain a clear understanding of the patient’s medication experience.

### 4.4. Limitations

A limitation of inclusion criteria in this analysis is that the term medication experience is not often used directly. While the authors took measures to operationalize the agreed upon criteria for inclusion and exclusion, it is possible that some articles were missed. While this may be limiting, the authors believe that for the purposes of this analysis, sufficient articles were uncovered to contribute to our understanding. The ambulatory setting was purposefully selected to maintain a manageable scope for this study. Other types of settings, such as institutionalized in-patient care settings and long-term care facilities, were not explored here so we acknowledge this limitation. However, broader contexts could be potentially rich sources for future study of the medication experience.

## 5. Conclusions

The medication experience is an important concept for pharmaceutical care. The definition of the medication experience put forward in this analysis provides a solid building block for beginning theory development. The concept analysis also provides a conceptual definition for research that has implications for operationalizing the concept of the medication experience in studies. The elements of attributes, related and surrogate terms, antecedents, and consequences have been consistently identified across interdisciplinary literature, adding greater breadth and depth to better understand the concept of the medication experience. It is hoped that this concept analysis will provide a solid foundation for theory development, research, and practice for pharmacists and other healthcare professionals.

## Figures and Tables

**Table 1:** Literature Search Strategy (July–August 2020).

Database	Search Terms and Strategies	Number of Articles
Ovid Medline	Medication experience.mp OR	84
	(medication*adj3experience*).ti OR	113
	(Medication* or medicine*) AND experience*.ti AND patient*.ti	284
	exp QUALITATIVE RESEARCH/or qualitative.mp. AND exp*Medication Adherence/AND patient*.ti	246
	Meaning of Medications	134
CINAHL	Medication Experience.ti	91
	Medicine Experience.ti	24
	Meaning of Medication	134
PsycINFO	Medication * Experience.mp	50
	Medicine * Experience.mp	18
	Meaning of Medic*.mp	85
Sociological Abstracts	Medication Experience.ti	15
	Medicine Experience.ti	64
	Meaning of Medication	143
Google Scholar	Medication Experience (allintitle)	66
	Medicine Experience (allintitle)	161
	Meaning of Medication (allintitle)	49

**Table 2:** Social constructions of medical terms common to the patient encounter regarding the medication experience.

Term	Provider Perspective (Biomedical or Clinical)	Patient Perspective (Lived Experience)
Insight	Into physiological, biomedical knowledge and how treatment works, i.e., one has a disease or diagnosis for which medications are needed	How the patient experiences living with the illness and medications—one has hope for a better life and resilience
Adverse effect	Biomedical impact to an objective body	No longer feeling like one’s self or vulnerable to social construction of stigma
Benefit or purpose of medication	Long-term control of disease or symptomatic relief	Pragmatic experience of patient. Sense of control over one’s life; being able to do the things in life one wants to do
Expert	Of science-informed knowledge of biomedicine and clinical expertise	Of living in one’s own body and life experience and what gives their life meaning and quality
Medication adherence	An (over)simplified endpoint or goal	A means to a goal, a part of a journey of recovery or healing that involves struggle and requires skill building

**Table 3:** Summary of attributes.

Attribute	Elements of the Attributes
1. Ambivalence	a. resistance b. necessary evil c. cost and benefit
2. Vulnerability	a. perceived and actual effect of drug on body b. long-term use c. reliance/dependence on healthcare system and providers d. reliance/dependence on communication and information
3. Socially Constructed	a. medications as symbols b. norms, perceptions, beliefs c. social environment influence d. healthcare context and biomedicine e. sense of self
4. Pragmatic	a. ability to evaluate from the patient perspective b. priority of wanting to feel well c. barriers to everyday living d. practicalities of medication use
5. Contextual and nuanced	a. illness experience and health context b. daily life circumstances c. specific medications d. personal beliefs/attitudes/desire for involvement
6. Active ongoing process	a. resistance and acceptance b. evaluative process c. control and self-regulation d. process that takes time and has no end e. burdensome and requires effort

**Table 4:** **Exemplar:** Taken from “Local discourse on antiretrovirals and the lived experience of women living with HIV/AIDS in Thailand” by Liamputtong et al., 2015, two excerpts that describes the medication experience with taking anti-retroviral medication for HIV/AIDS [37].

Excerpt	Attribute
(1) “Due to the fear of side effects of (anti-retroviral) ARVs, one woman was reluctant to take them” [37] (p. 259).	*AMBIVALENCE*
(2) “ARVs have provided hope for HIV-positive people.	
	*SOCIALLY CONSTRUCTED*
ARVs act as part of ‘people’s ‘quests’ to regain control, create order,	*ACTIVE ONGOING PROCESS*
reduce dependence on others	*VULNERABILITY*
and to feel ‘normal’ again’ [76] (p.376).	*SOCIALLY CONSTRUCTED*
However, as Nguyen et al. (2007) suggest, it is crucial to pay attention to how individuals experience and deal with the challenges associated with ARVs in their everyday life	*PRAGMATIC*
	*ACTIVE ONGOING PROCESS*
within a local context” [77], ([37] p. 260).	
	*CONTEXUAL AND NUANCED*

**Table 5:** Attribute inquiry examples (or patient interview questions) for practitioners relevant to the patient’s medication experience.

Attribute	Specific Inquiry Examples (Patient Interview Questions)
**Ambivalence**	How is both wanting to take medicine but not wanting to take medication because of personal and socio-cultural issues expressed?
**Vulnerability**	What feelings about medications are involved? (i.e., hope/hopelessness, self) What systems or power dynamics complicate their situation such as the medical system, access to treatment, trust in provider to help them understand their health?
**Socially Constructed**	How has the patient’s sociocultural reality been constructed such as influence of culture, environment, media, family and friends, and issues based on past events and policy?
**Pragmatic**	What are the concrete aspects of this person’s reality for medication-taking?
**Contextual and Nuanced**	How does the context and nuances of the patient’s social/cultural/historical/psychological/physical illness inform the patient perspective and decisions made?
**Active Ongoing Process**	How does the patient seem to be evaluating the use of medications such as feelings of stigma, routines, if medication is needed, implications for social life and cost?
